# Thermal Management in Metal Hydride Hydrogen Storage Reactors: Mechanisms, Architectures, and Design Trade-Offs

**DOI:** 10.3390/nano16050303

**Published:** 2026-02-27

**Authors:** Quanhui Hou, Xiao Xu, Ke Deng, Yuchen Li, Qianyang Wang, Zhihao Xu, Jiayu Ji, Yunxuan Zhou, Zhao Ding

**Affiliations:** 1 School of Automotive Engineering, Yancheng Institute of Technology, Yancheng 224051, China; 2 College of Materials Science and Engineering, National Engineering Research Center for Magnesium Alloys, National Innovation Center for Industry-Education Integration of Energy Storage Technology, Chongqing University, Chongqing 400044, China; 3 Lanxi Magnesium Materials Research Institute, Lanxi 321100, China

**Keywords:** solid-state hydrogen storage, thermal management, hydride bed heat transfer, reactor, reactor architecture, design trade-offs

## Abstract

Metal hydride-based hydrogen storage reactors combine high volumetric hydrogen density with intrinsic safety, yet their performance is fundamentally limited by inefficient thermal management arising from the strong coupling among heat transfer, thermodynamics, and reaction kinetics. The highly exothermic and endothermic nature of hydrogen absorption and desorption requires rapid and spatially uniform heat removal or supply, which is difficult to achieve due to the low thermal conductivity and complex internal structure of hydride beds. This review presents a mechanistic and architectural overview of thermal management in metal hydride hydrogen storage reactors. Key heat transfer limitations within hydride beds are first analyzed, followed by a systematic classification and critical comparison of major thermal management architectures, including bed-level modifications, structural reactor designs, and heat-exchanger intensification strategies such as embedded tubes, fins, and phase-change materials. The advantages and limitations of these approaches are discussed in terms of heat transfer efficiency, hydrogen storage capacity, structural complexity, and scalability. Finally, the review highlights the central design trade-offs governing compactness, efficiency, and manufacturability, and outlines future directions toward application-oriented and scalable reactor design through integrated thermal and structural optimization.

## 1. Introduction

Hydrogen energy, as a clean, efficient and renewable energy carrier, plays a crucial role in addressing global climate change, achieving energy structure transformation, and building a sustainable society. Its unique high energy density and the fact that its combustion products are only water make it an ideal substitute for fossil fuels [1,2]. However, the wide application and large-scale promotion of hydrogen are largely constrained by issues of storage and transportation [3,4]. Compared with existing energy carriers, the low density characteristic and high flammability and explosiveness of hydrogen make efficient, safe and economical hydrogen storage technologies the focus and bottleneck of current energy research [5,6,7].

Currently, the mainstream methods of hydrogen storage include high-pressure gaseous hydrogen storage [8,9], low-temperature liquid hydrogen storage [10,11,12], solid-state hydrogen storage [13,14,15], and organic liquid hydrogen storage [16,17,18], etc. Among the various hydrogen storage technologies, solid-state hydrogen storage based on hydrogen storage materials is regarded as one of the most promising solutions due to its advantages such as high hydrogen storage density, good safety, and mild operating conditions [13,14]. Solid-state hydrogen storage media such as metal hydrides have the advantages of high hydrogen storage density, good chemical stability, good reversibility, and high safety [19,20]. However, during the actual hydrogen absorption and release process, there will be intense energy release and absorption phenomena. Their inherent low thermal conductivity and limited pore connectivity also lead to poor heat and mass transfer performance, making it difficult for the advantages at the material level to be fully exerted at the system level [21,22,23,24].

Therefore, the design of the hydrogen storage reactor is of vital importance for enhancing the hydrogen absorption and release performance [25,26]. During the hydrogen absorption process, if the large amount of reaction heat released in a short period of time cannot be promptly conducted to the outside of the reactor, it will inhibit the progress of the hydrogen absorption reaction and reduce the reaction efficiency. During the hydrogen release process, if heat cannot be promptly provided to the interior of the reactor, the hydrogen release reaction cannot proceed [27,28,29].

The hydrogen storage reactor, as the core equipment for holding and storing hydrogen, its design, material selection, manufacturing process, and system integration directly determine the overall performance and feasibility of hydrogen storage technology. However, existing hydrogen storage reactors still encounter many challenges in practical applications. Although solid-state hydrogen storage materials can store hydrogen at normal temperature and pressure, their hydrogen storage density, charging and discharging rates, and cycle life still need to be further improved, and the integration and management of hydrogen storage reactors also have complexity [30,31]. Therefore, continuously optimizing the materials and design of hydrogen storage reactors is the key to achieving efficient, safe, and economical hydrogen storage and application.

This review aims to systematically summarize the current research status and development trends of the structural and performance optimization of hydrogen storage reactors. Compared with other reviews on hydrogen storage vessels, this review presents a systematic investigation into vessel structures and heat transfer structures from the perspective of the overall development of metal hydride reactors. It imposes a rigorous classification and hierarchical progression on the optimization of metal hydride beds and various heat transfer structures, and enumerates innovative and composite structures. Meanwhile, it compiles and visualizes key performance indicators to provide a clear comparative framework and practical pathway for subsequent research and engineering implementation. By summarizing and analyzing the simulation and experimental results from the existing literature, we identified the common patterns of the response behaviors of hydrogen storage reactors under different operating conditions and design parameters, and evaluate and compare the applicability and effectiveness of various optimization methods. We focused on the heat transfer characteristics of hydrogen storage reactors, the progress in bed layer optimization, the research progress in reactor structure design, material selection, structural innovation, and lightweight design. We also paid attention to the development of advanced simulation technologies and the optimization of reactor heat exchange structure design. Through this comprehensive review, this paper hopes to provide valuable references for researchers in related fields, with the aim of accelerating the research and industrialization process of advanced hydrogen storage technologies and providing theoretical support for the development and commercialization progress of hydrogen storage reactors.

## 2. Working Principle and Heat Transfer Characteristics of Solid-State Hydrogen Storage

### 2.1. Principle of Hydrogen Storage in Metal Hydrides

Metal hydride (MH) storage refers to the reversible chemical reaction of hydrogen with metals and alloys to form stable metal hydrides (such as LaNi_5_H_6_, MgH_2_, etc.). Hydrogen is held in chemical bonds within the material’s crystal lattice [32,33]. This mechanism has high hydrogen storage capacity, strong stability, and is significantly safer than high-pressure gaseous hydrogen storage. However, the reaction process is accompanied by significant thermal effects, and it is necessary to break chemical bonds through temperature control to achieve the release and storage of hydrogen [34,35].

The MH hydrogen absorption and release process can be expressed by the following reversible reaction equation:

M + H_2_ ⇌ MH_x_ + Δ*H* (1)

The reaction enthalpy Δ*H* determines the heat release per unit amount of substance during the solid-state hydrogen storage process, and it forms the thermodynamic basis of the thermal effect. The larger the absolute value of the reaction enthalpy, the stronger the heat release will be at the same reaction rate.

**Figure 1:**
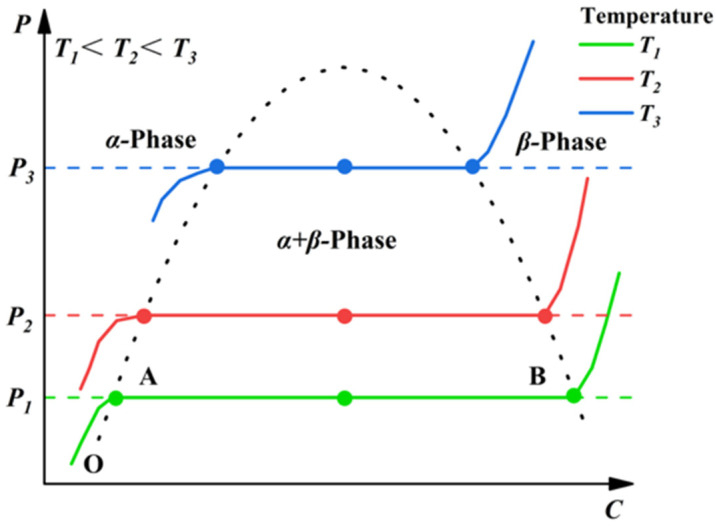
Schematic diagram of the P–C–T curve for solid-state hydrogen storage alloys.

The thermodynamic characteristics of this hydrogen absorption and release reaction process can be tested and studied through the pressure composition isotherm (P–C–T) [36,37]. Under equilibrium conditions where the α-phase and β-phase coexist, the P–C–T curve exhibits a plateau region. Under equilibrium conditions where the α-phase and β-phase coexist, the P–C–T curve exhibits a plateau region, as illustrated in Figure 1. Here, the horizontal axis C denotes the hydrogen storage capacity, while the vertical axis P represents the equilibrium hydrogen pressure. Taking temperature T_1_ as an example, the hydrogen absorption process comprises three distinct stages.

(1) Starting from point O, as the hydrogen pressure in the system increases, hydrogen gradually diffuses from the metal surface into the interior of the metal, thereby forming a solid solution, and the curve extends to point A.

(2) As the hydrogen pressure continues to rise, hydrogen reacts further with the solid solution, and the alloy is in a state where the MH phase and the solid solution phase coexist. The curve in segment AB is usually a gentle straight line; this region is also called the plateau region, and the corresponding pressure P_1_ at this stage is called the plateau pressure.

(3) With a further increase in hydrogen pressure, the hydrogen storage alloy reaches a state of hydrogen absorption saturation, can no longer absorb additional hydrogen, and the hydrogen absorption reaction terminates. At this point, the alloy no longer absorbs hydrogen, and the hydrogen pressure in the system rises significantly.

The equilibrium pressure P can be calculated using the Van ‘t Hoff formula [38,39], as shown in Formula (2):

(2) $$\ln\frac{P}{P_{ref}}=\frac{∆H}{RT}-\frac{∆S}{R}$$

where *P*—Equilibrium pressure, Pa (the hydrogen absorption pressure balance and hydrogen release pressure balance are represented by *P_A_* and *P_D_*, respectively.). ∆*H*—Reaction enthalpy, J/mol; ∆*S*—Reaction entropy, J/(mol·K); R—Gas constant; T-Temperature, K; P_ref_—Reference pressure, 0.1 MPa.

During the hydrogen absorption and release process of metal hydrides, temperature and hydrogen gas pressure are the main factors influencing the reaction rate. The expressions for the hydrogen absorption rate and the hydrogen release rate are respectively shown in Formulas (3) and (4) [40]. This formula needs to satisfy the following necessary assumptions: ① The porous MH reaction bed area has local thermal equilibrium characteristics. ② For the MH reaction bed, the effects of heat radiation and convective heat transfer of the fluid are ignored in the heat transfer analysis. ③ The hydrogen in the reactor is regarded as an ideal gas with uniform pressure distribution. ④ The thermophysical properties of the hydrogen storage material were considered to be constant.

(3) $${\left(\frac{dX}{dt}\right)}_{a}=C_{a}exp\left(-\frac{E_{a}}{RT}\right)\ln\left(\frac{P_{g}}{P_{A}}\right)(1-X)$$

(4) $${\left(\frac{dX}{dt}\right)}_{d}=C_{d}exp\left(-\frac{E_{d}}{RT}\right)\frac{P_{g}-P_{D}}{P_{D}}X$$

where $\dot{m_{H}}$—Rate of change of hydrogen density during the hydrogen absorption process, kg/(m^3^·s); C_a_—Hydrogen absorption constant, 1/s; C_d_—Dehydrogenation constant, 1/s; *E_a_*—Activation energy of the hydrogen absorption reaction, J/mol; *E_d_*—Activation energy of the dehydrogenation reaction, J/mol; R—Universal gas constant, 8.314 J/(mol·K); *T*—Average temperature of the reaction bed, K; *P_g_*—Hydrogen pressure, Pa; *P_A_*—The equilibrium hydrogen pressure of the material during the hydrogen absorption process, Pa; *P_D_*—The equilibrium hydrogen pressure of the material during the hydrogen dehydrogenation process, Pa; *X*—Metal hydride reaction fraction.

From Formulas (3) and (4), it can be seen that temperature has an impact on both the hydrogen absorption and desorption rates. However, since the absorption process is exothermic, the generated thermal energy must be efficiently dissipated from the hydride bed. Conversely, hydrogen desorption is an endothermic process that necessitates external heat input to drive the reaction. Consequently, the augmentation of heat transfer performance is deemed a critical factor in optimizing the overall kinetics of both hydrogen absorption and desorption [41].

### 2.2. Heat Transfer Characteristics of Hydrogen Storage Reactors

**Figure 2:**
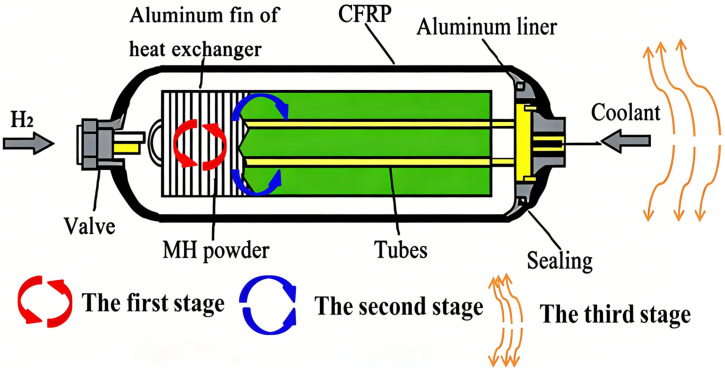
Schematic diagram of the heat exchange process in a hydrogen storage reactor.

A solid-state hydrogen storage reactor consists of a reactor shell, a hydrogen storage bed, a heat transfer structure, a heat transfer medium, a gas path, and other components. Based on the heat transfer mechanism, the heat exchange process is generally divided into three stages, as illustrated in Figure 2. (1) Heat transfer within the hydrogen storage material, where the primary influencing factor is the thermal conductivity of the material [42]. Some researchers have designed a fin-metal foam reactor with a thermal resistance as low as 0.0099 K/W [43]. (2) Heat exchange between the hydrogen storage material and the heat exchanger, where the primary influencing factors are the contact area between the hydrogen storage material and the wall surface, as well as the spatial distribution of the contact interface. The magnitude of the contact area determines the heat flux between the hydrogen storage material and the wall, whereas the spatial distribution of the contact interface affects the uniformity of the temperature distribution within the hydrogen storage material during the hydrogen absorption and desorption processes [44,45]. For instance, Bai, C. et al. [46] employed a topology optimization approach to optimize the heat dissipation fins. In comparison to the initial metal hydride tank, the modified tank equipped with optimized fins demonstrated an 86.1% reduction in hydrogen absorption time. (3) Heat exchange between the heat exchanger and the external environment, which is primarily governed by the properties of the heat transfer medium.

Different hydrogen storage materials have different thermal conductivities and reaction rates, which lead to different constraints in different heat transfer stages during practical applications. Table 1 lists the thermal conductivity and other parameters of different hydrogen storage materials, which helps us determine the heat transfer limitations of the system under typical operating conditions.

Hydrogen storage reactor directly restricts the hydrogen absorption and release rate and system energy efficiency. Its heat transfer characteristics are as follows.

(1) The temperature field of the reactor should be changed, and changes in the temperature field can reversely regulate the hydrogen absorption and release rate by affecting the reaction equilibrium pressure and activation energy. This coupling relationship makes the system prone to hysteresis effects. For example, the local temperature rise caused by the heat release of hydrogen absorption will increase the equilibrium pressure and inhibit the phase change reaction; the temperature drop caused by the heat release of hydrogen release will reduce the reaction kinetic rate [52,53].

(2) The heat transfer path is complex and multidimensional. Heat transfer in the reactor involves multi-media and multi-mode coupling, including: convective heat transfer between hydrogen and hydrogen storage material particles, heat conduction within and between hydrogen storage material particles, contact heat transfer between hydrogen storage material, and reactor wall/heat exchange structure; some enhanced designs also include phase change heat transfer of phase change materials (PCMs) [54].

(3) The temperature field exhibits significant stratification. Due to the low thermal conductivity of hydrogen storage materials (e.g., the effective thermal conductivity of MgH_2_ is only 0.48 W/(m·K)), the reaction heat cannot be transferred rapidly, resulting in obvious temperature stratification within the reactor. Lewis, S.D. et al. [55] employed a serpentine flow field radiator, achieving superior heat transfer performance and faster hydrogen storage capability, which significantly improved the uniformity of the temperature field.

## 3. Research Progress in Solid-State Hydrogen Storage Reactors

### 3.1. Optimization of the Bed in Solid-State Hydrogen Storage Reactors

During the hydrogen absorption/desorption cycling of metal hydrides, the repeated expansion and contraction of the crystal lattice leads to pulverization of the hydrogen storage alloy particles, which in turn reduces the effective thermal conductivity of the reaction bed. Introducing thermally conductive additives such as metal foam, graphene, and carbon nanotubes into the reaction bed can effectively improve its effective thermal conductivity and enhance the heat transfer uniformity of the metal hydride reactor.

Shaji, S. et al. [56] designed a composite MH bed embedded with lead foam, which not only enhanced heat transfer to improve the adsorption performance of the hydrogen storage vessel but also mitigated homogenization-induced vessel strain by controlling the spatial variation of foam density. In finned heat transfer structures, the synergistic effect between the fins and metal foam is significant; it has been demonstrated that the same heat transfer performance can be achieved by adding only 50% metal foam [57]. The addition of expanded natural graphite (ENG) can also enhance the thermal conductivity of the MH bed. An optimization distribution method based on the extremum principle of heat transfer dissipation has been applied to the performance optimization of MH reactors. A multi-layered disc bed is divided into three zones, where the mass fraction of ENG decreases sequentially from the inner layer to the outer layer. Specifically, the optimal configuration allocates 20% ENG in the innermost layer, 12.6% in the middle layer, and 5% in the outermost layer. Consequently, the gravimetric energy output rate is improved by 8.22% compared to a uniform configuration [58]. Building upon this research, Liu, X. et al. [41] developed a composite material formed by mixing hydrogen storage alloys with expanded graphite, which enables synergistic heat exchange with the heat transfer fluid. The composite MH bed demonstrated superior performance in removing reaction heat and reducing bed temperature, thereby enhancing the uniformity of the temperature distribution within the reaction bed and shortening the hydrogen absorption reaction time by 49.5%. Furthermore, studies involving the simultaneous addition of expanded graphite and metal foam have revealed significant improvements in heat transfer rates, reductions in hydrogen charging time, and notable extensions in stable discharge duration [59].

To address the issue of deteriorating heat transfer capability in traditional MH reactors with increasing height, Xu, H. et al. [60] proposed a method to optimize the thermal conductivity of metal foam with a graded porosity. When the uniform porosity of the metal foam was 0.91, the hydrogenation time was reduced from 210 min to 193 min compared to a uniform porosity metal foam of the same volume ratio, demonstrating a higher reaction rate. Regarding bed nanostructuring, Selimefendigil et al. [61] incorporated TiO_2_ as a nanomaterial catalyst into the MH bed to enhance thermal management during the hydrogen absorption process, thereby improving the hydrogen storage kinetics of the MH reactor. The high specific surface area in nanostructures can shorten the diffusion path of hydrogen atoms and reduce the dynamic barriers and thermodynamic stability of hydrogen storage materials, thereby enhancing their performance [62].

In summary, the incorporation of thermally conductive materials such as metal foam into the MH bed can improve the rates of hydrogen absorption and desorption; however, it also exerts a non-negligible impact on the overall hydrogen storage capacity. Consequently, one of the key priorities for future research is to develop strategies that minimize the usage of conductive additives while simultaneously maintaining the requisite thermal conductivity.

### 3.2. Reactor Structure Design

(1) Tubular reactor

Tubular reactors were the first to be researched and developed. A tubular reactor consists of a shell and a hydrogen tube in the center. MH fills the annular space between the hydrogen tube and the outer wall of the reactor. It has good sealing performance and can withstand high pressure. The height-to-diameter ratio of a tubular reactor is usually greater than 10. This slender structure gives it excellent heat exchange performance. However, the large height-to-diameter ratio of tubular reactors restricts their overall height, resulting in a relatively low basic hydrogen storage capacity. Although the hydrogen storage performance can be improved by modifying the metal hydride bed, the available storage space is inherently fixed, leading to only a limited enhancement in capacity. Furthermore, optimizing the heat transfer structure to improve its heat transfer performance will further compress the installation space for the metal hydride bed, resulting in a non-negligible trade-off between heat transfer performance and storage capacity.

**Figure 3:**
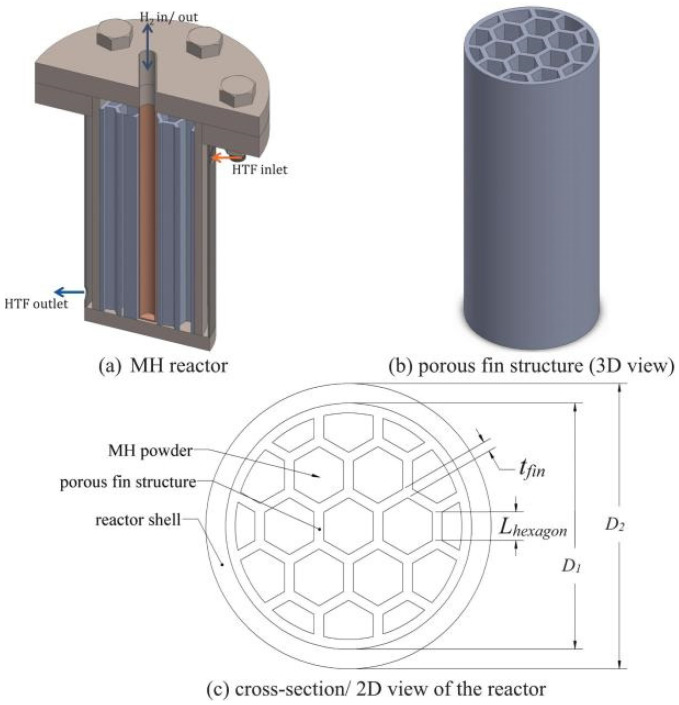
The geometric structure, external morphology, and planar cross-sectional views of the honeycomb-like straight tube structure [66]. Copyright 2026 Elsevier.

In the early stages, Lee, S.G. et al. [63] investigated a Zr-based hydrogen storage alloy reactor consisting of 14 independent copper tubular reactors. The heat transfer mode adopted was air cooling, with aluminum fins attached to the outer walls of the copper tubes to enhance heat dissipation capacity. Building upon this work, Ji, L. et al. [64] utilized longitudinal plate-shaped aluminum fins on the inner tubes and added a semi-tube helical coil around the vessel periphery to strengthen external heat exchange, thereby achieving more uniform thermal management of the entire container. With the continuous advancement of technology, novel tubular reactor configurations have emerged, such as the multi-tube shell-and-tube heat exchange vessel. This design combines multiple straight tubes separated by baffles within a shell structure. Except for the straight tubes and baffles, the interior of the shell is filled with cooling fluid. The upper and lower ports of the vessel are designed as inlets and outlets for the cooling liquid, and longitudinal fins are inserted inside the straight tubes to assist heat transfer. Compared to the discontinuous cooling fluid-air heat exchange employed in outer-layer designs, this cooling fluid-encapsulated heat transfer method is significantly more effective [65]. Notably, Jenne, S.P. et al. [66] proposed an innovative design to improve the mainstream straight-tube structure commonly adopted in tubular configurations, as shown in Figure 3. They used porous honeycomb finned ribs to partition the internal space of the reactor, which are dispersed from the central heat-exchange straight tube to the peripheral regions as the core. Meanwhile, the metal hydride was filled in segments, and a certain headspace was reserved to accommodate the expansion of metal hydride powder. Unlike conventional heat exchange with a single concentric tube, the honeycomb vertical spaces partitioned by the porous honeycomb finned ribs inside the vessel enable the segmented filling of metal hydride, which effectively isolates the central heat source and mitigates the poor heat transfer performance caused by the concentrated heat dissipation of the metal hydride bed. In the meantime, the thermal management of peripheral regions, which are usually overlooked in standard straight tubes, is integrated into the overall thermal control system of the vessel, resulting in a more uniform heat transfer distribution inside the reactor. This structure features a vertical configuration and is designed for practical applications with an emphasis on space efficiency, which is applicable to refrigerated fuel cell vehicles. It addresses the issue that the free expansion of metal hydride powder at the bottom exerts excessive stress on the reaction wall in the application of conventional vertical metal hydride vessels. When this structure operates in an alternating heating and cooling mode within the metal hydride system of fuel cell vehicles, its performance can be fully utilized to maintain hydrogen supply and system cooling. For the cooling system of a 10 kW heavy-duty refrigerated transport truck, the required replacement frequency can be reduced by up to 48% at a hydrogen flow rate of 1 g/s and by 63% at a low flow rate of 0.2 g/s with a fin volume fraction of 21%, which significantly extends the service life of the reactor, so the recycling efficiency has been effectively enhanced. Meanwhile, the straight tubes separated by the porous honeycomb demonstrate remarkable manufacturing convenience; they can be applied to most vertical metal hydride vessels simply by adjusting the pore size. Due to its simple structure, its manufacturing cost is much lower than that of other complex heat exchange structures.

(2) Disc reactor

The reaction bed of the disk reactor is disk-shaped. In the traditional layout, hydrogen is introduced from one side of the disk reactor and heat is exchanged on the other side. The disk reactor has a small hydrogen storage capacity, thin reaction layer, large heat exchange area, and good reaction performance [67,68]. However, since the upper and lower sides of the reactor need to pass hydrogen and exchange heat respectively, the hydrogen storage capacity cannot be increased by increasing the number of reactor units.

Yang, X. et al. [69] constructed a three-dimensional model of a microchannel reactor equipped with a jacketed disc heat exchanger to investigate the effects of pressure-swing hydrogen storage on reactor heat transfer and reaction efficiency. Under operating conditions with a pressure step of 0.25 MPa and a time step of 14 s, the reactor exhibited the optimal performance, with the metal hydride bed achieving an average temperature of 627.13 K and a maximum temperature of 660 K.

(3) Tank reactor

Tank-type hydrogen storage reactors have become a research hotspot due to their large internal cavity, which allows for a higher loading of metal hydrides and thus greater hydrogen storage capacity [47,70,71]. Early tank reactors depended primarily on heat transfer through the outer wall. Due to the large volume of the internal metal hydride bed, this external-to-internal heat transfer mode yielded low heat exchange efficiency and non-uniform internal temperature distribution. Jemni, A. et al. [72] designed a fixed tank reactor utilizing external cooling. This reactor employed water bath heat exchange through the outer shell, which suffers from a limited heat exchange area and low efficiency. As heat is conducted through the reactor wall, the bed layer near the wall cools first during the initial stage of the reaction, while the central region remains difficult to cool and maintains a high temperature, leading to uneven temperature distribution.

**Figure 4:**
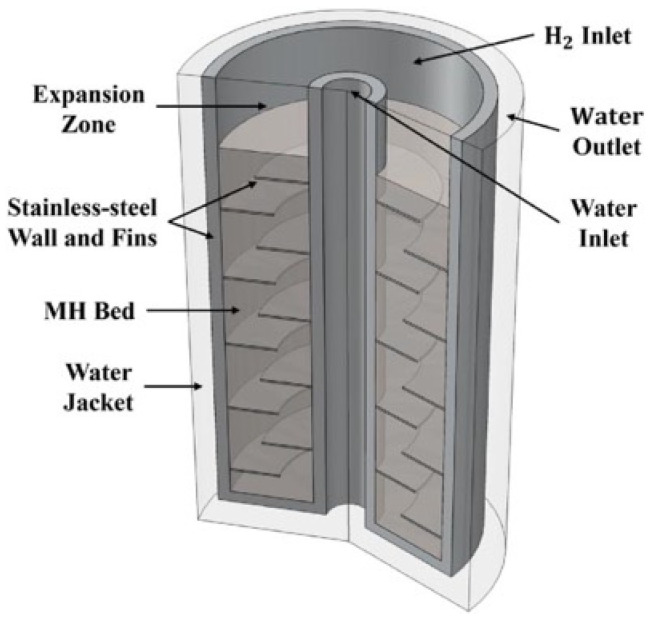
Geometric appearance of the MH vessel featuring water pipes with staggered fin arrangement and a water jacket [75]. Copyright 2025 Elsevier.

Building upon this, integrating fins on the external heat transfer fluid side of the reactor can reduce the time required to achieve 90% hydrogen charging by 10% [73]. Moreover, the adoption of heat dissipation fins can further improve the overall heat dissipation performance [74]. Dae Yeob Lee et al. [75] synthesized previous research findings by developing a hybrid design. They added fins to the outer side of the inner wall of the gas storage tank for heat dissipation and also incorporated fins around the central cooling flow tube. Additionally, they retained the traditional external heat exchange structure by wrapping the exterior with a water jacket, as illustrated in Figure 4. This combination of multiple heat exchange mechanisms significantly improved heat transfer and increased the reaction rate of the vessel. Precision cooling offers a more optimal solution for reactors [76].

Wang, J. et al. [77] added targeted delivery tubes inside a cylindrical activated carbon-based hydrogen adsorption tank to achieve the precise delivery of cooling gas to specific regions. By combining precooled gas with additional heat dissipation fins, the time required to reach an adsorption mass of 0.05 kg was reduced by 792 s compared to the original tank, and the total storage mass was increased by 27.7% at the end of the charging process.

Different hydrogen storage reactor structures have advantages and disadvantages, as shown in Table 2. In the future, it is an important research trend to explore the structural matching of lightweight shells and high filling rate beds to solve the contradiction between large capacity and uniform heat exchange. The integration of advantages from various cross structures can provide valuable references. The slender geometry and efficient heat transfer characteristics of tubular structures can be adopted to optimize the internal flow channels of the reactor tank. Furthermore, combining these with the thin bed design of disc-type structures can improve the edge thermal management of tubular components. Ultimately, we can achieve a comprehensive breakthrough in structural miniaturization, precise heat exchange, and efficient hydrogen storage to meet the practical application needs of hydrogen energy storage.

### 3.3. Optimization of Reactor Heat Exchange Structure Design

(1) Straight tube

A heat transfer tube (HTT) embedded in regions where heat tends to accumulate can effectively control temperature rise and enhance the reaction kinetics. They also have strong adaptability to the bed, wide application in actual production, and potential for large-scale manufacturing, so they have attracted widespread attention since the early stages of heat transfer structure development. At present, the development of straight-tube structures mainly falls into three categories: (1) multi-straight-tube systems, including general hydrogen storage reactors composed of multiple straight tubes and micro-multichannel hydrogen storage reactors; (2) combined heat transfer structures introduce additional heat transfer components such as helical tubes and fins, thereby further enhancing the heat transfer efficiency of straight tubes; and (3) geometric modification of straight tubes.

In the early stages, Xiang Y M. et al. [78] designed a rectangular microchannel reactor where multiple miniature straight tubes were arranged internally to expand the heat transfer area. This configuration demonstrated significant improvements compared to conventional disc and tubular reactors, enabling the hydrogen storage alloy to achieve 90% of its saturated hydrogen absorption capacity within 30 min. Studies have found that adding a cooling flow tube at the center of the vessel can enhance radial heat transfer and improve thermal output capability [79], providing a reference for the subsequent arrangement of multi-tube channels. For instance, in a honeycomb arrangement of multiple straight tubes, each tube mutually influences the others due to a butterfly effect, thereby enhancing the overall hydrogen storage efficiency of the vessel [80,81]. To balance vessel appearance, MH bed characteristics, hydrogen storage capacity, and efficiency for better large-scale industrial hydrogen storage, Wei Gao. et al. [82] optimized a multi-straight-tube hydrogen storage vessel using a multi-step numerical method. They proposed specific optimization schemes for metal foam porosity, volume ratio, and the number of heat exchange tubes. The optimized hydrogen absorption performance was 2.05 times higher than that before optimization.

The combination of straight tubes and fins is also a very common heat exchanger structure. Serge Nyallang Nyamsi et al. [83] added annular fins around the cooling fluid straight tubes in a hydrogen storage vessel to compensate for the drawback of heat easily concentrating in the central region in single straight tube heat exchange. By optimizing fin spacing and thickness, the hydrogen absorption reaction time was reduced by approximately 50% compared to a tube without fins, although a heat transfer blind zone at the bottom still exists [84]. Some scholars have also used conical fins to improve heat transfer performance [85]. Addressing the problem of heat dissipation blind zones easily occurring at the bottom, Wei Yang et al. [86] added curved fins in the opposite direction to conical fins inside a U-shaped tube, covering from the straight section to the bottom of the U-shaped tube. Compared with a single U-shaped tube, the hydrogen absorption reaction rate was nearly doubled. Other studies have demonstrated that densely wound disc fins can yield favorable heat transfer performance; however, this configuration compromises the inherent advantage of easy assembly and disassembly associated with shell-and-tube reactors [87].

**Figure 5:**
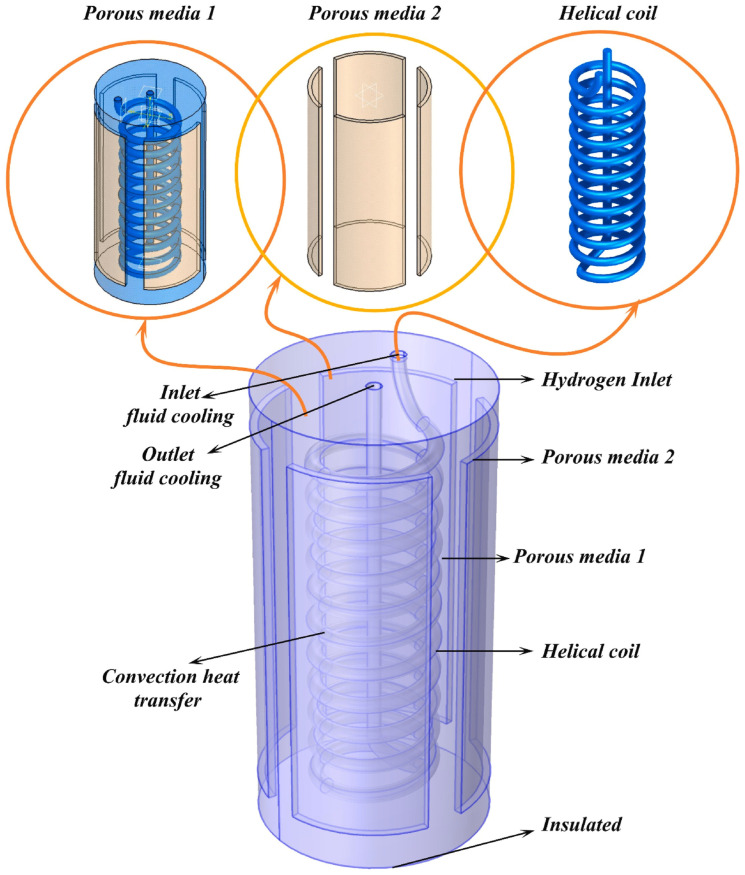
Geometry of the spiral straight tube return structure [89]. Copyright 2025 Elsevier.

In addition to fins, Xuan Huang et al. [88] investigated combined heat exchange using straight tubes and helical tubes. The combination improved the uniformity of the internal temperature field in the hydrogen storage tank, and they found that this configuration could achieve ideal temperature uniformity during both hydrogen charging and standing phases. As shown in Figure 5, splicing the lower end of a straight tube with the lower end of a helical tube can improve the utilization efficiency of the cooling fluid. Increasing the number of coil turns from 6 to 20 reduced the average bed temperature from 574.9 K to 542.8 K and increased the hydrogen concentration from 0.85 to 0.95 within 50 min. Increasing the coil pitch and angle can improve the convective heat transfer coefficient by up to 281%, but it also increases the pressure drop by 268% [89]. The manufacturing difficulty of this structure is only concentrated at the bottom of the connection between the spiral tube and the straight tube. Both manufacturing convenience and performance are improved simultaneously. Furthermore, the cooling effect introduced by the bottom reflux structure can effectively reduce the operating temperature of the pipeline, enhance the service life of the composite structure, and lower the replacement frequency, thereby further reducing the costs.

(2) Helical tube

Compared with straight-tube structures, helical-tube structures have a larger heat transfer area and better axial elasticity, which help to eliminate thermal and mechanical stresses caused by temperature changes and MH volume expansion.

In the early stage of spiral tube technology development, S. Mellouli et al. [90] designed a spiral tube heat exchanger with a gas charging/discharging port at one end. Due to the curved structure of the tube body, centrifugal force is generated when fluid flows through the pipeline. The secondary flow induced by this centrifugal force significantly enhances the heat transfer efficiency, thereby optimizing the overall performance of the vessel. On this basis, experts and scholars shifted their research focus from radially arranged spiral tubes to axially extended helical tubes, which feature more diverse structural forms and greater optimization potential, and a series of studies have been conducted to demonstrate their feasibility. Subsequently, P.V. Jithu et al. [91] conducted a detailed investigation into the feasibility of using helical tubes as MH metal beds. They placed a spring-like helical tube inside a directional vessel and observed various parameters during hydrogen injection. The hydrogen storage material begins to react and expand upon the introduction of hydrogen, and the heat of adsorption released during this process dissipates into the gas stream. The driving element, namely the spring helical tube, responds to temperature changes in the air duct through the displacement of its free end [92].

**Figure 6:**
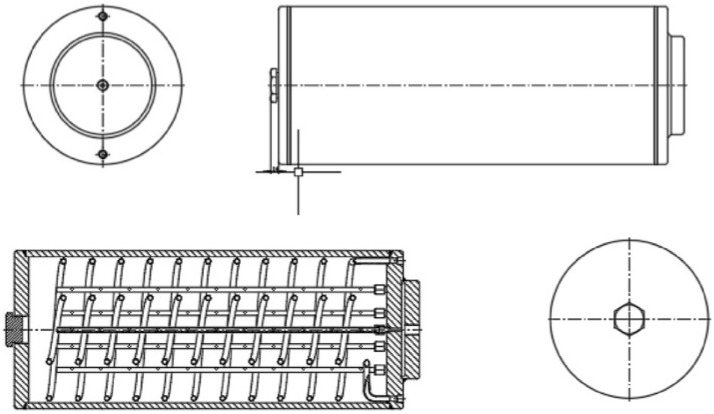
Structural model of the spiral microchannel reactor [97]. Copyright 2022 Elsevier.

Zhen Wu et al. [93] performed a performance comparison between a helical tube structure, a traditional straight tube structure, and a finned straight tube structure in experiments. The results indicated that the reactor employing the helical tube heat exchanger exhibited superior heat and mass transfer performance for the same surface area, attributed to the secondary circulation characteristics of the helical tube. Specifically, the dehydrogenation time of the helical tube reactor was reduced by approximately 26% compared to the straight tube reactor. A comparative analysis of the heat transfer performance and hydrodynamic characteristics between helical and straight tube structures revealed that under all tested Reynolds number conditions, the helical coil design consistently achieved a 8–12% higher Nusselt number than the straight tube configuration while significantly reducing the outlet temperature [94]. When comparing helical coil heat exchangers with different coil pitch sizes and curvature ratios to double-tube heat exchangers containing straight tubes, the helical coil exhibited a friction factor approximately 97% higher. However, leveraging the effects of larger pitch sizes, curvature ratios, and secondary flow, the Nusselt number increased by 34% [95]. Some scholars have combined straight tubes with helical tubes, embedding a straight tube at the center of the coil heat exchanger to address the heat dissipation blind zone at the center of the helical tube, thereby further expanding the effective area for central heat exchange [96]. On this basis, progress has also been made in the optimization of the helical tube’s own geometric parameters (such as tube diameter). Jianguang Yuan et al. [97] designed a Spiral Microchannel Reactor (SMCR), as illustrated in Figure 6. This structure effectively increases the contact area between the heat exchange tubes and the hydrogen storage bed by uniformly arranging spiral heat exchange channels along the axial direction of the vessel. Numerical simulations were conducted to investigate the effects of heat exchange tube diameters (10–25 mm) on the thermal behavior during hydrogen absorption and desorption. The results demonstrated that increasing the tube diameter significantly reduced the temperature at the bed center and accelerated the reaction rate.

Building upon numerous studies, the structural optimization of helical tubes primarily focuses on the optimization of liquid flow direction and the addition of heat exchangers. Amir Hossein Eisapour et al. [98] incorporated a straight tube into a traditional helical tube structure and connected the straight tube to the bottom of the helical tube to form a return flow configuration, with both the inlet and outlet located at the same end. The combined design of the helical heat exchanger and the central return tube significantly enhances the heat transfer efficiency between the cooling fluid and the metal alloy, reduces the bed temperature, and thereby improves the hydrogen absorption rate. When the helical coil heat exchanger and the central return tube are properly configured, the hydrogen absorption reaction time can be shortened by 24%. Applying this return flow structure to the middle section of the helical tube creates a countercurrent flow at the node where the helical tube conveys downward. The overall heat transfer coefficient of the heat exchanger equipped with a helical flow reverser is increased by 39.5% to 49.9% compared to a straight helical tube heat exchanger, representing a substantial improvement in heat transfer performance [99].

Despite the development of such return flow and countercurrent structures, research on parallel configurations has never ceased. Siyu Zheng et al. [100] arranged the inlet and outlet ports of the helical tube in a square container to be parallel and on the same side (and parallel to the ground). Studies have shown that with an appropriate helical pitch, this parallel natural convection effect can be effectively enhanced, while the thermal boundary layer on the tube wall is weakened, thereby improving the heat transfer performance [101]. In addition to conventional flow path designs, Gloria Biswal et al. [102] proposed a conical helical tube structure. This novel configuration regulates the heat transfer characteristics and flow dynamics under varying operating parameters, offering a new strategy for the innovative design of helical tube reactors.

Many scholars have also focused their research on double helical tube designs. The double helical design proposed by P. Mohamed Jaseel et al. [103] is capable of storing 14.07 kg of LaNi_5_ and absorbing 90% of its hydrogen filling capacity within 251 s. Adding rectangular fins to the double helical return tube for external fin reinforcement can effectively improve heat transfer in the surrounding regions [104]. Furthermore, the fins facilitate a uniform distribution of the fluid over the coil surface, thereby enhancing the heat transfer efficiency [105].

In recent years, with the continuous development of science and technology, a variety of innovative optimization strategies have been proposed and applied. Jinxiang Sun et al. [106] utilized a genetic algorithm and response surface methodology for the multi-objective optimization of the heat dissipation performance of helical tubes. Compared to the original design, the optimized heat transfer coefficient was increased by 15%, and the pressure drop on the tube side was reduced by 50.76%. Additionally, machine learning prediction models can be employed to optimize the layout of helical coils [107].

(3) Fin

Tubular heat exchange tubes (especially straight tubes) have a relatively small contact area with the MH, resulting in low heat transfer efficiency during the heat exchange process between the tubes and the MH. Increasing the number of heat exchange tubes can improve the heat transfer rate to a certain extent, but it reduces the filling amount of hydrogen storage materials. Fins possess advantages such as a simple structure, large heat transfer area, and high heat transfer efficiency [108,109]. The versatile design flexibility inherent in fins provides significant potential for the development of innovative heat exchange structures. Consequently, finned heat exchangers have been widely employed in the thermal management systems of hydrogen storage reactors.

Different structural forms of fins exert distinct effects on hydrogen storage performance. H. Chang et al. [22] concluded that the reaction performance of MH reactors with helical fins is superior to that of reactors with longitudinal fins. Furthermore, the larger the fin spiral cycle (SC), the faster the reaction rate. When the fin spiral cycle increases from 1/4 to 1, the hydrogenation time and dehydrogenation time can be reduced by up to 29.8% and 29.2%, respectively, while the hydrogen storage density remains unchanged. When the fin number (FN) increases from 4 to 16, the hydrogenation time and dehydrogenation time can be reduced by 53.1% and 34.7%, respectively, although the hydrogen storage density decreases by 24.1%. For helical fins, increasing the fin length (FL) or spiral angle (SA) helps to expand the heat exchange surface, thereby improving the heat transfer efficiency and accelerating hydrogen absorption [110]. Studies have found that the influence of fins on heat transfer becomes more pronounced when the thermal conductivity of the fins is increased relative to the bed, when the spacing between fins is reduced, and when the fin usage is increased [111]. Anurag Singh et al. [112] perforated ordinary annular radial fins to reduce concentrated heat dissipation and decrease the mass ratio of the heat exchanger assembly within the vessel to achieve a higher hydrogen storage capacity. Under conditions of a supply pressure of 15 bar, a coolant temperature of 298 K, and a flow rate of 1 m/s, hydrogen absorption was completed in 18 min. Compared with conventional fins, the perforated annular radial fins not only inherit the easy-to-manufacture advantage of annular fins, but also improve the heat dissipation effect, further increase the hydrogen storage capacity, and reduce the storage cost of hydrogen storage vessels, showing promising prospects for application and promotion. In addition, their scalability is greatly enhanced. Conventional fins can generally only be welded in fixed heat exchange structures or vessels and cannot be replaced. In contrast, the perforations in these fins allow for modular assembly in disc-type vessels, making them much easier to disassemble. Moreover, considerable room remains for further optimization of the specific parameters of these fins. Chao Bai et al. [62] designed a continuous strip fin structure where the strip fins are inclined at 45 degrees. These strips all start from the cooling boundary and are uniformly distributed inside the heat transfer system. The arrangement towards the storage tank inlet allows gas to rapidly infiltrate the metal bed upon entering the hydrogen storage tank. Through the combination of the high thermal conductivity of the material and the unique continuous strip structure, heat can be transferred efficiently inside the hydrogen storage vessel, resulting in a more uniform temperature distribution. Compared to an unoptimized hydrogen storage tank, the charging time is shortened by approximately 86.1%.

**Figure 7:**
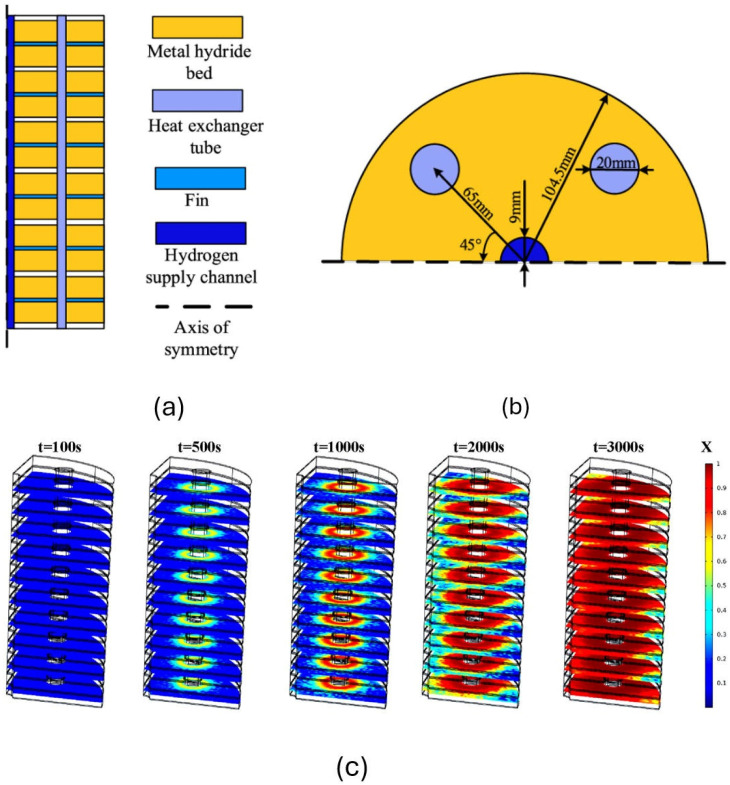
(**a**) Metal hydride reactor vessel with finned straight tubes. (**b**) A half-section view of the vessel. (**c**) Distribution of the reacted fraction in the MHB at different times [113]. Copyright 2023 American Chemical Society.

The Weishu Wang team employed finned and heat exchange tube technologies, which significantly improved the heat transfer efficiency of the magnesium hydride energy storage system. Figure 7a shows the metal hydride reactor vessel with finned straight tubes, and Figure 7b presents a half-section view of this vessel. Figure 7c illustrates the distribution of the reaction zone within the magnesium hydride bed (MHB) at different times under tube heat dissipation. Hydrogen storage initially occurs near the heat transfer fluid (HTF). As time progresses, a ring-shaped structure gradually forms around the HTF and expands outward until it reaches the vessel wall, completing the storage process. It can be observed that the addition of fins has mitigated the shortcomings of the original single straight tube, which suffered from uneven heat transfer, resulting in slow and non-uniform hydrogen storage. With fins, a significant hydrogen storage capacity is also achieved in the edge regions [113].

Bionic principles are also frequently applied in fin design research. K Venkata Krishna et al. [114] designed a bionic leaf-vein fin structure. Through an optimized design featuring a 7 degree inclination angle and 4 fins, 90% storage capacity was achieved in only 57 s, representing a 73% reduction in absorption time compared to a single-tube reactor with longitudinal strip fins. Compared to traditional longitudinal fins, this narrow trapezoidal channel design with closely connected adjacent fins provides superior heat transfer performance. Building upon bionic structures, some scholars have optimized tree-shaped fins based on entropy theory using Response Surface Methodology (RSM) and mass transfer theory. The optimized fin structure exhibited a 11.5% higher Gravimetric Entropy Output Rate (GEOR) than the unoptimized tree-shaped fin structure [115]. Additionally, other researchers have employed topology optimization methods to redesign tree-shaped fins. Compared to conventional longitudinal fins, the time required to reach 90% hydrogen storage capacity was reduced by nearly 52% [116]. Furthermore, Mikiyas Hluf Hailekiros et al. [117] designed a novel multi-branched tree-like fin structure with a relatively simple configuration. This structure allows for the adjustment of the number of fin branches and angles according to different metal reaction beds and hydrogen charging/discharging environments, demonstrating strong production adaptability. Studies have shown that the double-branched tree-shaped fins significantly improved the thermal uniformity and reaction kinetics, reducing the complete hydrogenation time from 3617 s (longitudinal) and 3231 s (single-branched) to 2846 s.

**Figure 8:**
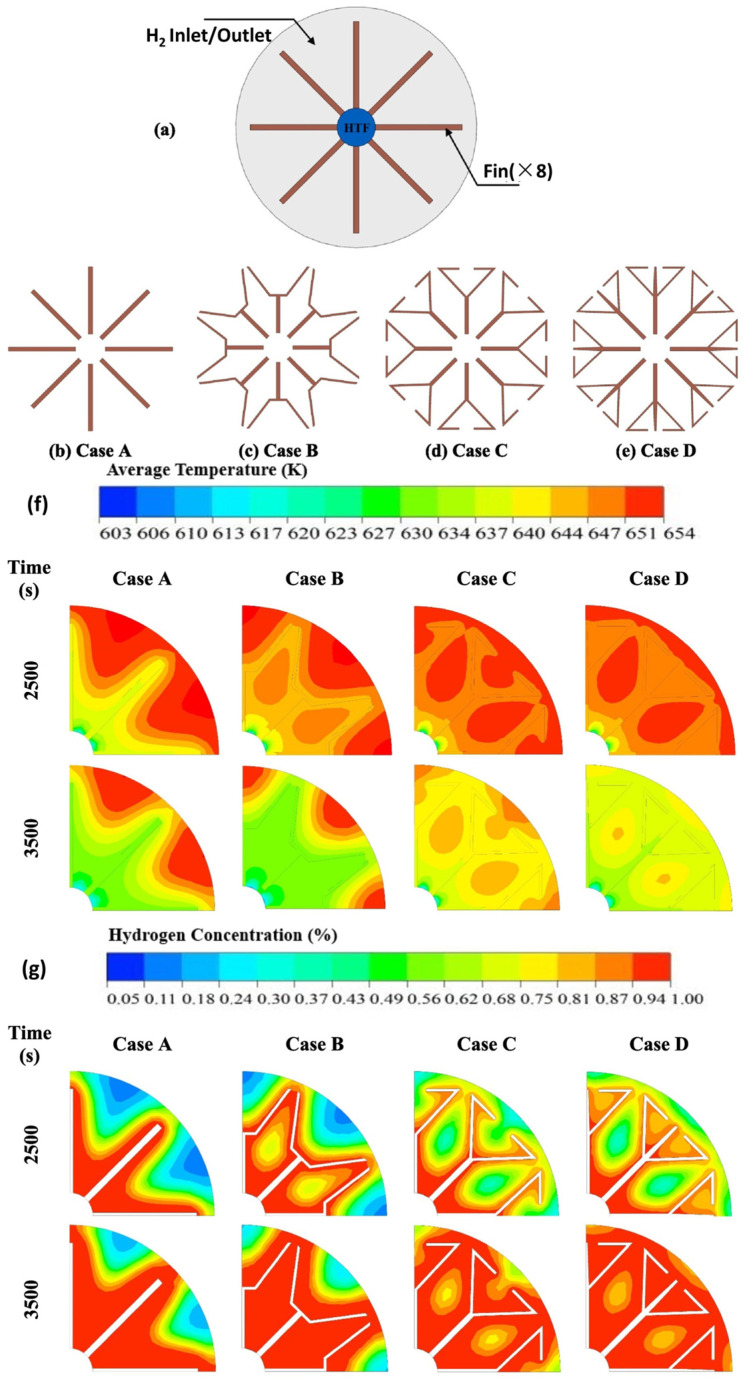
The average temperature and hydrogen concentration of the metal hydride (MH) bed and the MH bed fins at 2500 s and 3500 s after the initiation of hydrogen absorption, corresponding to the hydrogen storage tank and fin design. (**a**) Schematic diagram of MHSS with heat exchanger tube incorporating fins, (**b**) quadrilateral fin (Case A), (**c**) Y-shaped fin design I (Case B), (**d**) Y-shaped fin design II (Case C), and (**e**) triple-branched fin design (Case D); (**f**) average temperature, and (**g**) hydrogen concentration [118]. Copyright 2025 Elsevier.

Recently, a novel trifurcated fin structure was designed based on the Y-shaped fin structure [118]. Figure 8 illustrates the structural configurations of four different fin types. Figure 8a shows a top cross-sectional view of the vessel with finned heat exchanger tubes. Figure 8b depicts a quadrilateral fin (Case A), Figure 8c shows a Y-shaped fin design I (Case B), Figure 8d presents a Y-shaped fin design II (Case C), and Figure 8e illustrates a trifurcated fin design (Case D).

Figure 8f,g illustrates the temperature contours and hydrogen concentration contours along the MHHS (Metal Hydride Heat Sink/Storage) for different fin designs. The figures show selected times after the start of hydrogen absorption, including 2500 s and 3500 s. Significant differences in temperature variation among the four cases can be observed at 3500 s. As seen in Cases A and B, the temperature of the MH bed is relatively high, particularly in the outer regions. In contrast, the MH bed temperature in Case D is more uniform and significantly lower than in the other cases. It is evident that increasing the fin surface area from the Y-shaped fins (Case B) improves the internal heat transfer performance of the MHHS, especially when using the trifurcated branched fins (Case D). The hydrogen concentration contours in Figure 8b support this fact, as higher hydrogen concentrations are typically observed around the heat pipes and fin regions.

(4) Other complex structures

In recent years, 3D modeling and simulation tools have advanced considerably. Aiming to improve the hydrogen absorption performance of reactors and the temperature uniformity within metal hydride beds, researchers have dedicated increasing efforts to the design of more sophisticated reactor configurations. Building upon the research of heat exchange structures such as straight tubes, helical tubes, and fins, numerous scholars have conducted comprehensive combinatorial studies and innovations by integrating the advantages of these various structures [119].

Xiao Shuai Bai et al. [120] constructed a multi-heat-exchange MH reactor using annular finned tubes and a jacketed cooling system. A cooling fluid inlet was positioned at the center of the vessel, and two cooling fluid outlets were installed on the vessel wall. A layer of annular fins was added around the central cooling fluid inlet tube. Four hydrogen inlet tubes were arranged axially symmetrically near the central cooling fluid inlet tube, with each hydrogen inlet tube also equipped with fins. The remaining space within the vessel was filled with MH. The time required for this reactor configuration to reach 90% hydrogen saturation was reduced by 56.8% and 81.9% compared to a cylindrical MH reactor with a finned double U-tube heat exchanger and a cylindrical MH reactor with a finned single-tube heat exchanger, respectively. Heat exchangers can also adopt a double-layer design, where a large number of U-tubes form parallel pipelines, and curved fins and double rows of longitudinal fins are added around the U-tubes. The heat exchange fluid passes through the numerous inner and outer U-tubes and is then discharged through a coil at the bottom of the vessel. The curved fin structure of the heat exchanger can be bent at specific angles as needed to optimize space utilization and enhance heat transfer efficiency [121]. Wenyan Bi et al. [122] modified one end of a general cylindrical vessel into a conical shape and replaced traditional straight tubes with bellows arranged in a square distribution. This conical port is well-suited to the heat transfer direction in the bellows. Further optimization of the heat transfer area of the bellows and the heat transfer area per unit gap volume was performed using the Monte Carlo algorithm. The hydrogen absorption performance at 560 s reached 1.36 times that of ordinary straight tubes. Inspired by bionic principles, a bionic scroll disk reactor was designed by combining a general disk reactor with a helical tube, while adding phase change materials (PCMs) in the gap regions within the reactor to further improve heat transfer performance. In this configuration, the close integration between the metal hydride (MH) bed and the hydrogen inlet region effectively shortens the heat transfer distance. Furthermore, tuning the tube length and the number of layers enables the scroll structure to demonstrate outstanding stability and scalability, which significantly improves its adaptability to various operating scenarios and the controllability of hydrogen storage capacity [123].

Shell-and-tube reactors are also a popular field for the application of various heat exchange structures due to their larger heat transfer surface area to volume ratio and ease of manufacturing, disassembly, and use. Gayatri Kuchi et al. [124] designed a simple high-temperature shell-and-tube straight-tube heat exchanger with four ports: shell inlets/outlets and fluid inlets/outlets. This heat exchange relies mainly on the contact between the flow direction of the vessel fluid and the vessel wall. Its advantage lies in its combinable use during hydrogen production and storage, as well as its ease of convenient manufacturing and placement. Building upon the traditional shell-and-tube reactor, the hydrogen inlet end of the vessel was designed to be semicircular, and the original device inlet on the outer shell of the shell-and-tube reactor was changed to a cooling fluid inlet. The interior of the vessel contains multiple MH tubes. A reactor with 37 tubes reached 90% of its maximum hydrogen storage capacity within 535 s, reducing the absorption time by 80.3% and 49.3% compared to reactors with 7 tubes and 19 tubes, respectively [125].

**Figure 9:**
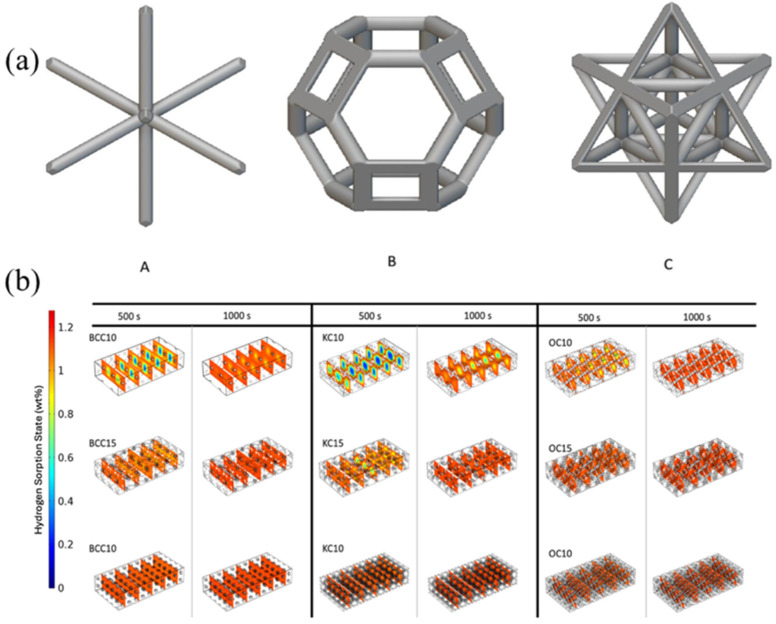
(**a**) Isometric views of a single cell of A (BBC), B (KC), and C (OC). (**b**) Hydrogen-charged distribution map of each structure after 500 and 1000 s [127] (the numbers following BCC, KC, and OC represent the cell size). Copyright 2025 Elsevier.

Inspired by this multi-tube shell-and-tube concept, Andrea Ambrosino et al. [126] incorporated lattice geometry to design a cylindrical hydrogen storage tank featuring an internal grid lattice with integrated pipe distribution. Although the vessel possesses only one large central cooling fluid channel, the grid structure filled with MH exhibits extremely high density, dividing the large central heat source into numerous small heat source units. This reduces heat propagation losses to a low level within a simple structure. Different from this simple lattice filling structure, Luthfan Adhy Lesmana et al. [127] also investigated more complex internal lattice structures. They selected representative structures from BCC (body-centered cubic), KC (Kelvin cell), and OC (octet cells) for study, as shown in Figure 9a. The hydrogen distribution diagrams of different structures at 500 s and 1000 s are shown in Figure 9b. The OC structure exhibited a more uniform absorption front advancing gradually from the boundary to the interior. OC15 maintained better uniformity and had fewer local slow absorption regions than the BCC and KC lattice structures, while avoiding higher stress and deformation. In contrast, larger cell sizes, such as 20 mm, showed obvious internal gradients, indicating slower and less efficient hydrogen adsorption.

Inspired by the improved heat transfer directionality of shell-and-tube reactors, J. Sunku Prasad et al. [128] designed an annular tank composed of three concentric nested tubes. The MH alloy is filled in the annular space between the inner and middle tubes. Cooling and heating of the annular MH bed are achieved by circulating HTF through the inner tube and the annular space between the middle and outer tubes. The cooling fluid outlet is positioned at the lower port in the direction of fluid circulation for discharge. Additionally, annular internal radial fins are added around the concentric tubes to enhance heat exchange. The team placed the cooling fluid inlet and outlet tubes on the same side, creating a rectangular circulation pattern for the fluid flow in the cross-section of the vessel interior. This configuration further reduces the heat loss caused by heat transfer to the vessel wall [129].

Currently, a variety of heat exchanger configurations have been developed, yet issues such as insufficient heat exchange area and non-uniform temperature distribution still persist. These limitations need to be addressed through structural optimization. Approaches including fin reinforcement, metal foam filling, and honeycomb partition structures can be adopted to enhance the heat exchange contact area and the effective thermal conductivity of the reaction bed. Furthermore, for applications such as vehicle-mounted and distributed energy storage systems, stacked reaction beds and integrated heat exchange jackets have been developed to strike a balance between heat exchange efficiency and hydrogen storage density within constrained spatial conditions.

### 3.4. Phase Change Material (PCM)

Phase change materials (PCMs) are substances capable of absorbing and releasing heat during phase transition cycles. The use of latent heat from phase change energy storage materials for thermal management has found applications in various fields. Consequently, scholars have proposed a novel thermal management approach for reactors using PCMs, integrating them into MH reactors as heat transfer and storage media. Through the heating-melting process of PCMs during hydrogen absorption and the cooling-solidification process during hydrogen desorption, the transfer, storage, and release of reaction heat are achieved. It is evident that the combination of PCM and MH represents a promising thermal management method [130,131].

Among the various development directions, the compact disc-type MH reactor has been a key area for PCM application. Yang Ye et al. [132] cut PCMs into compressed discs with the same diameter as the MH reaction bed and arranged them in a longitudinally alternating manner within the reactor. Compared to the traditional surrounding layout, the hydrogen storage efficiency was significantly improved, with hydrogen absorption and desorption efficiencies increasing by 77.8% and 58.8%, respectively. Notably, they pressed the MH into a disk shape and reserved appropriate gaps during the addition or filling of PCM. This measure prevents container failure caused by thermal expansion of the PCM or MH during heat transfer, thereby making the simulation results more consistent with practical applications. Such a gap-reserving approach provides a useful reference for the subsequent design of similar containers. Building upon this research, the team added copper fins and copper foam to both sides of the PCM compressed discs to further enhance the heat transfer performance of the PCM. Compared to the finless structure, adding ten sets of copper plates increased the average hydrogen absorption efficiency by 28% [133]. During the heating process from the initial temperature of 293 K to the equilibrium temperature of 346 K, adding only fins around the PCM reduced the hydrogen absorption completion time from 2121 s to 1623 s. The PCM melted faster, and the hydrogen absorption rate was increased by 30.7%. To overcome the heat transfer limitation of the container, composite metal foam was applied on the PCM side. Under the same heating conditions, the hydrogen absorption completion time was further reduced from 1623 s to 1315 s, with the reaction rate improved by 23.4%. When the porosity of the composite metal foam was slightly decreased (from 0.952 to 0.888), the temperature and hydrogen absorption curves almost overlapped. The structure maintained good stability, while the average reaction rate was increased by 1.6%. They also designed an innovative reactor by reducing the length of a conventional multi-tube array MH reactor and refilling the reduced area and the space around the tubes with PCM to form a new tank [27]. In addition, Siwoo Jung et al. [134] utilized a self-developed 2D CFD-based MH hydrogen storage reactor model to optimize the effective heat transfer surface area of copper plates embedded in the PCM and the MH reaction bed. Compared to the finless structure, the absorption and desorption reaction rates were increased by 94.9% and 92.9%, respectively.

**Figure 10:**
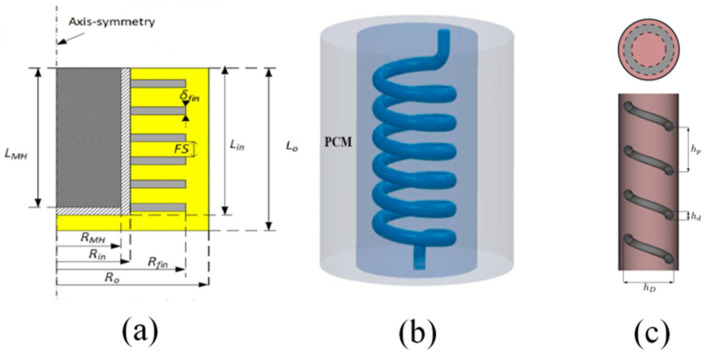
(**a**) Geometric structure of the modified configuration featuring external PCM with fins [135], (**b**) geometric structure of the combined helical HTF and PCM configuration [136], (**c**) geometric structure of the helical PCM composite configuration [137]. Copyright 2023 Elsevier. Copyright 2025 Elsevier.

Serge Nyallang Nyamsi et al. [135] focused their attention on the outer PCM, as shown in Figure 10a. They added fin structures to the PCM covering the container to improve its thermal conductivity. The strong heat transfer effect of the fins enabled the liquid-solid state transition of the PCM to occur more quickly, thereby accelerating the heat transfer rate within the container and enhancing the performance of the container. Wrapping the PCM around the outer periphery of the container effectively separates the MH from the PCM, which significantly reduces the risk of chemical reactions between them that could degrade the performance of the container. Furthermore, this configuration greatly mitigates the impact of PCM leakage during the melting process and maximally preserves the structural integrity and availability of the container. Figure 10b combined the PCM with a spiral HTF. The PCM stored 35.77% of the total reaction heat, while the spiral HTF, with a 37% larger heat transfer area and a 32% higher Reynolds number, achieved efficient heat removal and dissipated 64.2% of the reaction heat. The combined action of the two achieved a time to reach 90% hydrogen absorption capacity that was 48.2% shorter than that of the reactor equipped with only the spiral HTF [136]. Some researchers placed MH powder in the spiral tube and completely immersed it with two layers of PCM on the inside and outside, as shown in Figure 10c. Clearly, this fully utilized the advantages of the spiral tube and the phase change material, further expanding the heat surface area. Compared with the straight tube HTF structure with PCM, the absorption and dissociation time of hydrogen was shortened by 49% and 40%, respectively [137].

Adding fins to the outer PCM layer is a highly effective approach. Fins can be arranged in either an annular or longitudinal configuration. Analysis of the thermal profiles revealed that due to the natural convection effect of the molten PCM, longitudinal fin arrangements are more conducive to enhancing the hydrogen reaction rate within the vessel. Compared to the finless structure, the hydrogen absorption rate was increased by 60.91%, and the desorption rate was increased by 72.07% [138]. Although the influence of different fin arrangements on the liquid–solid phase state in the outer layer exists, scholars have pondered whether there is an underlying secondary reason. Talal Alqahtani et al. [139] innovated upon the traditional heat dissipation structure where the MH reactor is wrapped by a single layer of PCM. In the cylindrical annular sandwich structure they constructed, the external PCM wrapping was divided into inner and outer double layers, with the MH reactor sandwiched between them. This significantly improved the heat transfer efficiency, reducing the time consumption for hydrogenation and dehydrogenation by 81.5% and 73%, respectively. Inspired by the considerable improvement in MH reactor efficiency brought about by this simple variation in PCM filling layout, experts and scholars in the field have conducted more in-depth research into PCM filling arrangements. Hui Dai et al. [140] arranged PCM and the MH reaction bed in a fan-shaped layout to separate them and investigated the effect of the number of fan-shaped PCM partitions on the MH reactor in detail. Keeping the amounts of PCM and MH reaction bed constant, as the number of PCM partitions increased from 2 to 5, the 90% hydrogen absorption time was shortened from 2172 s to 518 s. Meanwhile, they also investigated the effect of different PCM masses in this structure. The results show that PCM performed stably under various mass conditions, and the temperature and reaction fraction curves maintained consistent trends, indicating that the phase change behavior and thermal transfer capability of PCM functioned normally. With the gradual increase in PCM mass, the corresponding time reduction rates were 8.69%, 6.77%, and 9%, respectively. However, it should be noted that the slight improvement in hydrogen storage rate accompanying the increase in PCM mass could not compensate for the reduction in volumetric/gravimetric hydrogen storage density, and thus did not yield a better overall positive effect; on the contrary, it reduced the durability of PCM. Therefore, selecting an appropriate PCM quantity is also an important aspect for further optimization of hydrogen storage vessels. Building upon the research of Hui Dai et al., the fan-shaped partitioned PCM and fan-shaped partitioned MH reaction bed were further layered and arranged in an alternating sequence with the MH reaction bed. This novel staggered arrangement significantly expanded the thermal contact area of the MH reaction bed, allowing for sufficient heat transfer between the PCM and the MH reaction bed. Compared to a general six-layer fan-shaped reactor, the hydrogen absorption time was reduced by 80.2% and 30.5% [141]. During the initial stage of the reaction, continuous exothermic hydrogen absorption increased the MH region temperature in both the six-layer sector reactor and the staggered structure reactor to approximately 346 K. The thermal gradient between the metal hydride and PCM regions drives directional heat transfer toward the PCM domain, activating both sensible and latent heat absorption via the dominant heat conduction mode. Sensible heat absorption prevails in the single solid-phase region, while heat conduction remains the primary heat transfer mechanism. Upon reaching the melting point of PCM, the phase change triggers substantial latent heat absorption, with heat conduction continuing as the main heat transfer pathway throughout the process, thereby gradually reducing the MH temperature. The staggered arrangement significantly enhances interfacial heat transfer efficiency compared with conventional designs, while ensuring more uniform PCM temperature distribution. Consequently, the PCM in the staggered reactor reaches its melting point earlier and achieves rapid heat absorption, demonstrating superior overall performance. It is evident that expanding the thermal contact area between the PCM and the MH reaction bed is the key to improving the performance of hydrogen storage vessels using PCMs.

Inspired by the above research, Ziyang Zhang et al. [53] designed a PCM filling layout featuring a well-shaped grid structure and a bionic spiderweb structure, arranged in an alternating annular pattern with the MH reaction bed. This divides a cylindrical MH reactor into multiple small units, resulting in a more uniform temperature distribution, further expanding the thermal contact area of the MH reaction bed and enhancing the heat transfer efficiency of the MH reactor. In comparison, the bionic spiderweb structure with the same number of 16 cells exhibited a 75.3% improvement in hydrogen absorption efficiency. Subsequently, a novel spiderweb structure was designed [142]. Compared to the bionic spiderweb structure designed by Ziyang Zhang et al., this structure fills the spiderweb fins, resulting in more cell distributions, a more compact annular layering, and more uniform filling. Compared to a finless MH reactor, the spiderweb fins can reduce the time required to reach the maximum hydrogen absorption capacity by 64.6%. When the number of annular fins is increased from 5 to 7, the hydrogen absorption time is shortened by 6.3%

Based on the research literature of various scholars, significant differences exist among different heat exchange structures in terms of heat transfer limitations, performance indicators, and design trade-offs. These differences directly determine the adaptability and superiority of each structure in different application scenarios. Combining existing research findings, the author systematically compared and analyzed four common heat exchange configurations: straight tube, spiral tube, finned structure, and phase change material (PCM), with detailed comparisons presented in Table 3. The straight tube serves as a basic heat exchange form due to its simple structure, convenient fabrication, and low maintenance cost. However, its heat transfer limitations mainly stem from limited heat exchange area and low heat transfer coefficient, requiring a fundamental trade-off between heat exchange efficiency and flow resistance in design. The spiral tube enhances flow disturbance through fluid rotation, improving the heat transfer coefficient and overcoming some limitations of straight tubes. Nevertheless, it suffers from high manufacturing difficulty and large pressure drop, necessitating a balance between heat transfer enhancement and energy consumption control. The finned structure effectively breaks through heat transfer limitations by expanding the heat exchange area, significantly improving heat transfer efficiency, but involves trade-offs including complex structure, increased flow resistance, and higher cost. Phase change materials achieve efficient thermal management through latent heat storage and release, addressing the constraint of unsteady transient heat transfer. However, they exhibit a slow heat transfer rate and large volume occupation, requiring a balance between thermal storage performance and heat transfer response speed in design.

## 4. Conclusions and Prospect

Solid-state hydrogen storage has a wide range of application prospects in the fields of new energy vehicles and energy storage due to its high hydrogen storage capacity and good safety. The hydrogen storage reactor of metal hydrides, as the core component of solid-state hydrogen storage, is particularly crucial. This paper summarizes the latest research progress in reaction bed optimization, reactor structure optimization, and heat exchanger design optimization. Different thermal management methods have their own advantages and disadvantages, and designing an efficient and compact thermal management system remains an object that requires continuous efforts and pursuit. Based on the current development trends of materials science and thermal management technology, the main development directions of metal hydride hydrogen storage reactors in future engineering applications are as follows:

(1) Research and development of high-performance metal hydride materials. The intrinsic properties of metal hydride materials are the core bottleneck restricting reactor engineering. In the future, it is necessary to focus on thermodynamic regulation, kinetic enhancement, and stability improvement as the core to achieve the matching of materials and operating conditions. Based on density functional theory (DFT) and molecular dynamics simulation, through multicomponent alloying and nano-composite modification technologies, the thermodynamic and kinetic parameters of hydrides are regulated.

(2) Multi-field coupling optimization of the thermal management system. The heat transfer efficiency of the thermal management system directly determines the hydrogen absorption and release kinetics process of the reactor and the energy utilization efficiency. In the future, it is necessary to break through the limitations of a single heat transfer mode and construct an integrated thermal management system under the coupling of temperature, concentration, and stress. Efficient heat exchange structures with coupled mass transfer can be developed, and a topology optimization design can be used to integrate the heat exchange channels with the reaction bed layer, achieving the synergistic enhancement of heat transfer and mass transfer processes.

(3) Integrated and modular design of the reactor and system. Engineering applications have strict requirements for the compactness, adaptability, and scalability of the reactor. It is necessary to focus on integrated integration and standardized modularization to achieve deep matching between structural design and application scenarios. Based on process simulation, multi-device collaborative integration optimization can be carried out, and integrated valve groups and integrated flow channel design can be adopted to reduce the length of connection pipelines and leakage points, improving the volumetric power ratio of the system.

## Figures and Tables

**Table 1:** Comparison of parameters of different hydrogen storage materials.

	Thermal Conductivity (W·m^−1^·K^−1^)	Hydrogen Absorption Reaction Rate Constant (s^−1^)	Hydrogen Release Reaction Rate Constant (s^−1^)	Porosity	Ref.
LaNi_5_	2	59.187	9.57	0.5	[47]
MgH_2_	0.28	10^10^	10	0.74	[48]
TiFe	1.6	60	10	0.5	[49]
Mg-Ni-La-2Mn	2	1.92 × 10^8^	-	0.6	[50]
Mg_2_Ni	3.36	175.07	-	0.5	[51]
ZrCo	2.81	0.03	-	0.6	[51]

**Table 2:** Performance comparison of different types of reactor structures.

Type	Advantages	Disadvantages
Tubular Reactor	1. Excellent sealing performance and high-pressure resistance. 2. Superior heat transfer performance. 3. Suitable for modular design. 4. Compact structure with high space utilization efficiency.	1. Limited storage capacity (constrained by the compatibility of the height-to-diameter ratio of the tubular structure with the heat transfer structure). 2. Relatively simple structure, leading to limited potential for performance enhancement of the storage vessel.
Disc Reactor	1. Thin reaction bed layer. 2. Large heat transfer surface area. 3. Facilitates easy disassembly and maintenance.	1. Small storage capacity, which cannot be effectively increased by simply adding more units. 2. Complex structure, making it difficult to control manufacturing costs.
Tank Reactor	1. High hydrogen storage capacity. 2. Strong heat transfer capability. 3. Versatile integration of heat exchange structures; comprehensive research results available.	1. Diverse structural configurations make it challenging to balance heat transfer performance and storage capacity. 2. A wide variety of materials are available for structural optimization, which remains largely unexplored.

**Table 3:** Overview of Each Heat Exchange Structure.

Type	Heat Transfer Limitations	Design Trade-Offs	Comprehensive Evaluation
Straight Tube Structure	Limited by the aspect ratio, the radial heat transfer within the bed is non-uniform; the convective heat transfer intensity of the fluid is insufficient, which easily leads to local heat transfer bottlenecks.	Its structure is decentralized, but the distribution form is relatively simple, featuring high overall convenience and ease of manufacturing; the hydrogen storage efficiency is low when used alone; for the straight tube design, its performance should be optimized while maintaining its simple structure.	The structure is relatively simple, and the heat exchange efficiency and hydrogen storage capacity of a single configuration are low. Significant improvements can be achieved through optimization of the filling layout, the combination of multiple straight tubes and other heat exchangers. In addition, it exhibits strong scalability and a wide range of adaptability and applications.
Helical Coil Structure	Fluid stagnation is prone to occur at the bends of helical coils, resulting in reduced local heat transfer efficiency; stress concentration in the tube body can easily lead to structural damage, which indirectly affects the long-term stability of heat transfer.	Its structure is relatively compact, especially when the parameters of the spiral tube and the multi-spiral arrangement are specifically optimized. However, the manufacturing cost and difficulty are relatively high, which poses certain challenges to the promotion of its application. Although the hydrogen storage efficiency is relatively high, it also sacrifices the internal space of the container. The structural design should be further optimized between improving hydrogen storage capacity and convenience.	The structure is relatively complex, but the heat exchange performance and hydrogen storage capacity are further improved. However, its compatibility with other heat exchangers is poor, and the excessively complex structure will lead to a decrease in the hydrogen storage capacity of the container itself.
Finned Structure	Thermal resistance exists in the heat conduction of fins, resulting in reduced heat transfer efficiency in the deep part of the bed; the complex structure leads to an increase in fluid flow resistance.	The structure is relatively dispersed, with high hydrogen storage efficiency and ease of fabrication. Reasonably designing the fin density can reduce space occupation. The core concept of structural design is to achieve optimal performance through appropriate arrangement in the actual container, rather than excessive material usage and increased cost.	It has diverse structural forms and a large selection space; it also exhibits strong scalability. As an auxiliary heat exchange structure, combining it with any heat exchange structure can effectively improve the heat exchange efficiency and hydrogen storage capacity of the hydrogen storage container.
Phase Change Material (PCM) Structure	Volume changes during the phase change process induce stress, which affects structural integrity; phase change characteristics degrade under low-temperature environments, leading to a reduction in heat transfer capability. Additionally, phase change materials are prone to aging after long-term use, which impairs the stability of heat transfer.	Its structure is relatively compact, which greatly improves hydrogen storage efficiency; the combined use of multiple phase change materials can further enhance this efficiency. In addition, phase change materials have a wide range of selections, low costs, and are easy to manufacture and promote. In the design, attention should be paid to the balance between the selection and combined use of phase change materials.	It features a simple structure with various filling layouts and can be combined with any heat exchanger, resulting in a wide range of applications. When used alone, it provides a relatively low hydrogen storage capacity but excellent heat transfer performance. When used in combination, both the hydrogen storage capacity and heat transfer performance can be further optimized and enhanced.

## Data Availability

No new data were created or analyzed in this study. Data sharing is not applicable to this article.
